# Single-use ureteroscopes in ectopic pelvic kidney stones

**DOI:** 10.25122/jml-2021-0251

**Published:** 2021

**Authors:** Bogdan Geavlete, Razvan Popescu, Dragos Georgescu, Petrisor Geavlete

**Affiliations:** 1. Sanador Hospital, Bucharest, Romania; 2. Department of Urology, Sf. Ioan Emergency Clinical Hospital, Bucharest, Romania

**Keywords:** single-use flexible ureteroscopy, ectopic pelvic kidney, renal stones, abnormal kidney, laser, stone-free

## Abstract

We analyzed the results of single-use flexible ureteroscopy (su-fURS) with the holmium laser in treating renal stones associated with ectopic pelvic kidney (EPK). The study retrospectively analyzed data of 11 patients diagnosed with EPK and stone disease who underwent su-fURS between May 2017 and November 2019. The analyzed surgical data included the mean operation time, stone-free and complication rates, as well as hospitalization period. Disposable digital flexible ureteroscopes were exclusively used. The mean age was 55, with a 1.2:1 male to female ratio. The mean stone burden was 30±9 mm (ranging from 17 to 49 mm). The mean calculi digitized surface area (DSA) was 299±56 mm^2^ (ranging from 170 to 597 mm^2^). A ureteral access sheath was used in all 11 patients, and holmium laser lithotripsy was performed (dusting mode parameters: low energy – 0.5J, high frequency – 50 Hz, long pulse; pop-corn mode: high energy >1 J, medium frequency– 10–50 Hz, long pulse; fragmenting mode: high energy >1 J, low frequency <10 Hz, short pulse). The average operative time was 78±19 minutes (ranging from 68 to 144 minutes). The stone-free status (residual fragments <3 mm) after one session was 60.1%, 84.1% after the second session, and 94.4% after the third session. The hospitalization period was 29 hours (ranging from 17 to 39 hours). The overall complications rate (according to the Clavien-Dindo system) was 19.7%. Therefore, su-fURS represents an effective therapeutic approach characterized by a remarkably high stone-free rate and few complications in EPK-associated calculi.

## Introduction

Minimal invasive surgery technique is continuously developing, establishing new approaches for different pathologies and completing the idea of personalized treatment with significant benefits. Nephrolithiasis is one of the most frequent diseases in a urology department, and several methods are nowadays available for sustaining rapid results with minimal risks for the diagnosed patients.

In particular cases of abnormal anatomy, associated lithiasis can be a difficult challenge, and proper surgical indications may be confusing. An ectopic pelvic kidney is an anomalous condition that originates from embryological development [1]. Reported incidence for this variety of pathology was estimated to be 1 to 220–3000 cases [2]. Renal drainage can be severely affected, and as a direct consequence, urolithiasis increases the risk of further complications [3, 4].

While stone access difficulties in an abnormal urinary tract were described in several studies, endourological treatment represented a challenging situation for urologists [2]. Shock wave lithotripsy (SWL) and percutaneous nephrolithotomy (PCNL) are considered good alternatives but often raise technical difficulties, and success defined by the stone-free rate is frequently lower than those performed on patients with normal anatomy [5, 6]. Other approaches such as robotic, laparoscopic pyelolithotomy, or laparoscopic-assisted PCNL were also described during past years.

Nowadays, retrograde intrarenal surgery gained more attention in treating stones, usually smaller than 2 cm according to the actual guidelines, and offers an appropriate alternative due to low complications and a high percentage of stone-free rate [7]. Over the years, much progress has been made which considerably improved surgical aspects. Some of these findings, like smaller caliber, increased deflection, more powerful laser sources, and high-quality cameras, made flexible ureteroscopy a solid option in accessing the pyelocaliceal system [8].

The most recent achievement in retrograde intrarenal surgery, the development of single-use ureteroscopes, represents a significant step in urology. Besides the technical advantages, which already demonstrated single-use scope’s superiority compared to reusable ones, this complete sterile procedure definitely decreases the risk of hospital-associated infections. Some studies also sustain the idea of cost-effectiveness in favor of disposable devices [9].

Actual trends suggest personalized treatment for every patient according to the associated pathology following the idea of fewer complications, improved results, less hospital stay, and rapid social reintegration.

Since flexible ureteroscopes already demonstrated their superiority compared to PCNL, SWL or laparoscopy even when accessing an abnormal urinary tract, this study aims to evaluate the advantages expressed in the safety and efficacy of single-use devices in challenging situations such as an ectopic pelvic kidney.

## Material and Methods

**Figure 1.**
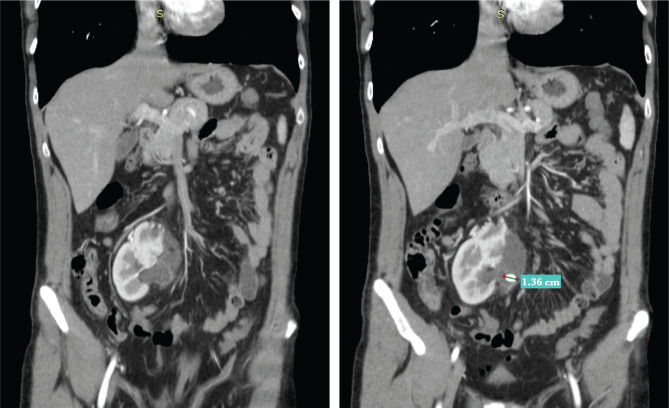
Coronal and sagittal CT sections of one case of ectopic pelvic kidney with duplicity successfully treated through single-use flexible ureteroscopy. The stone measured 1.36 cm on the longest diameter and the stone-free status was obtained after only one intervention.

**Figure 2.**
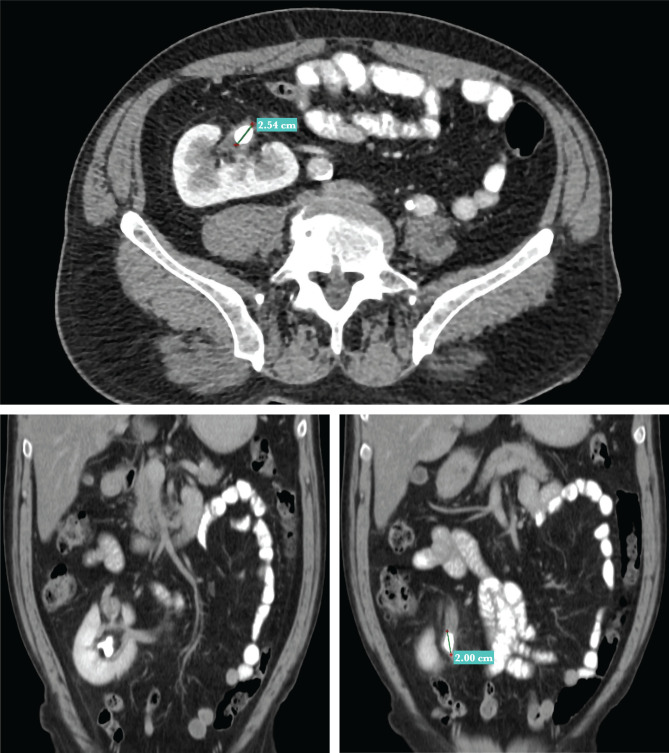
Coronal sagittal section. The longest diameter of the pelvic stone measures 2.54 and 2 cm.

Between May 2017 and November 2019, 11 patients diagnosed with calculi in ectopic pelvic kidneys in which single-use flexible ureteroscopy (sulfur) was performed were retrospectively analyzed. After searching the medical records, patients’ characteristics such as mean age and sex were noted. All patients were primarily assessed with complete blood tests previous to the intervention. Urine tests were mandatory, and positive urine cultures were adequately treated according to the antibiogram before intervention. The imagistic evaluation consisted of computed tomography (CT) scans (Figures 1 and 2) or intravenous pyelography (IVP); these tests were performed before the intervention, and several details such as stone size, location, hydronephrosis, or laterality were recorded.

The stone-free rate was evaluated based on a kidney, ureter, and bladder (KUB) X-ray and abdominal ultrasound evaluation of the urinary tract. Additionally, intraoperative and postoperative data regarding the mean operative time, average fluoroscopy time, number of sessions and complications rate using the Clavien-Dindo modified classification system were included in this study.

### Inclusion criteria

• Patients aged above 18 years old;
• Cases diagnosed with intrarenal lithiasis and pelvic ectopia;
• Surgical interventions performed with single-use ureteroscopes.

### Exclusion criteria:

• Pediatric patients;
• Patients with untreated coagulopathies;
• Other renal congenital abnormalities;
• Ureteral stone location;
• Stones located in a caliceal diverticulum.

### Retrograde intrarenal surgery (RIRS)

All the procedures were performed in a lithotomy position under spinal anesthesia using a single-use flexible digital ureteroscope manufactured by Zhuhai Pusen Medical Technology. In 36.36% of cases, a double J catheter was placed before the surgical approach. The PU3022 scope has a maximum diameter of 9.5 Fr, a working length of 650 mm, and a maximum deflection angle of 270 degrees at the distal tip. The working channel diameter is about 3.6 Fr. The optical specifications include 120 degrees field of view with 0 degrees line of vision and 3–50 mm visible range on the optical fiber. All 11 patients had a ureteral access sheath, and holmium laser fragmentation was performed using standard indications: for dusting-low energy: 0.5J, high frequency: 50 Hz, long pulse, for pop-corning-high energy: >1 J, medium frequency: 10–50 Hz, long pulse and for fragmenting-high energy: >1 J, low frequency: <10 Hz, short pulse.

The stone-free status was defined as residual fragments under 3 mm. Depending on the stone dimension, more than one session was necessary in several cases. A double J stent catheter was inserted at the end of each procedure. After achieving the stone-free status, the catheter was removed after 1 to 3 weeks, depending on the procedure complexity or complications.

### Data analysis

After collecting data from the medical records, we analyzed them using Microsoft Excel and Word available on Microsoft Office version 18.2008.12711.0.

## Results

Demographic details and stone characteristics are presented in Table 1 and Table 2. We included 11 patients diagnosed with ectopic pelvic kidney, including 2 cases of cross-fused ectopia and 1 case of pelvic duplicity. Regarding sex distribution, the study population comprised 6 males and 5 females, with an estimated ratio of 1.2:1 and a mean age of 55 years old. After analyzing the medical records, only 1 patient was diagnosed with chronic renal disease.

**Table 1.** Patients’ characteristics and stone aspects.

	**No. (mean)**		**Range**
**Age**	**55 (years)**		**29–71 (years)**
**Sex**	Male	Female	
	6	5	
**Stone size**	30±9 (mm)		17–49 (mm)
**Laterality**	Right	Left	
	9	2	

**Table 2.** Hydronephrosis and stone location.

	**No.**	**%**
**Hydronephrosis**		
No hydronephrosis	7	63.63
Grade 1 and 2	4	36.36
**Stone location**		
Renal pelvis	5	45.45
Caliceal	3	27.27
Staghorn	3	27.27

Details of previous endourological treatment for ureteral stones were noted in 2 cases. The mean stone burden was 30±9 mm, ranging from 17 to 49 mm^2^. The mean stone digitized surface area (DSA) was 299±56 mm^2^, ranging from 170 to 597 mm^2^.

**Figure 3.**
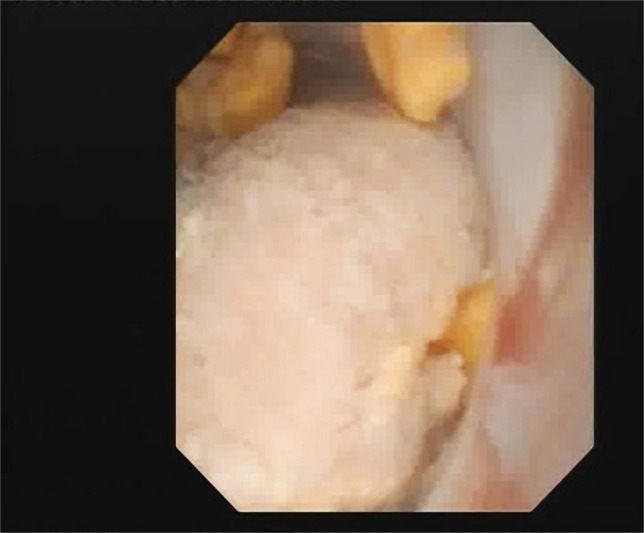
Ectopic kidney – multiple stones in the inferior calyx.

**Figure 4.**
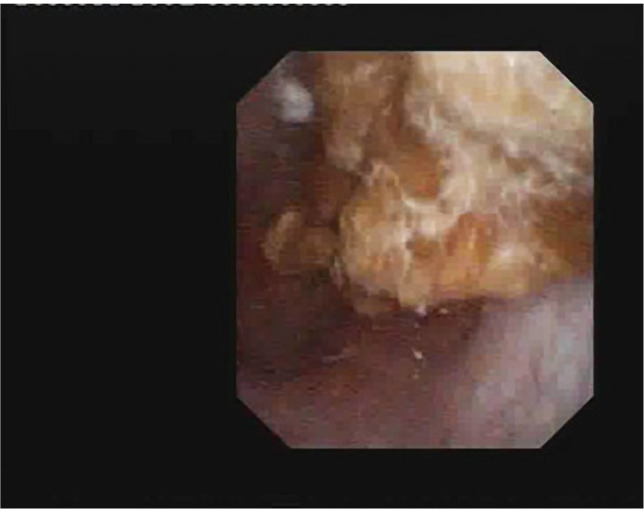
Ectopic kidney – pyelo-caliceal stone (pyelic and superior calyx locations).

Different stone locations and dimensions were one of the main factors which played an important role in the multiple interventions performed until the stone-free status was obtained (Figures 3 and 4).

Recorded data referring to operative details such as surgical time, fluoroscopy, and days of hospitalization calculated for each ureteroscopy session are shown in Table 3. When necessary, repeated ureteroscopy procedures were performed within one month from the previous intervention. The mean operative time was 78±19 minutes, with a slight decrease in length per session in cases that required reintervention for achieving the stone-free status. The average fluoroscopy time appeared to be increased when accessing the lower calyx as the stone location at this level was more difficult, but the overall X-ray exposure was about 58.9 seconds. Patients’ mean hospitalization time estimated per intervention respected the one-day surgery principle in most cases.

**Table 3.** Operative characteristics.

	**No.**	**Range**
**Operative time**	78±19 (min)	68-144 (min)
**Fluoroscopy**	58.9±12.3 (sec)	32-82 (sec)
**Hospitalization**	29±6 (hours)	17-39 (hours)

**Figure 5.**
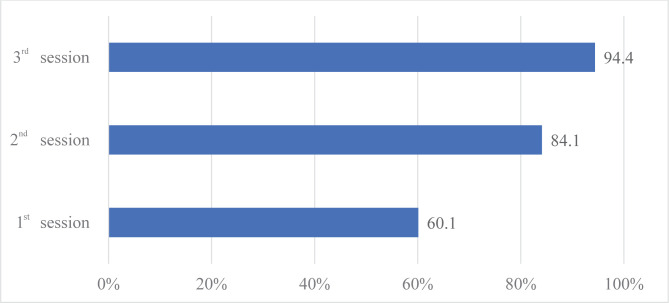
The stone-free rate related to the number of sessions.

This study included stones with dimensions from 17 to 49 mm, making more than one session of single-use flexible ureteroscopy necessary to obtain appropriate goals. As shown in Table 4 and Figure 5, the stone-free status was obtained after the first session in 60.1% of cases, while the percentage reached 94.4% after the third session. All 3 cases of staghorn calculi needed more than two interventions. During the procedure, no intervention was stopped for reasons such as technical failures, intraoperative complications, or decreased visibility while accessing the pyelocaliceal system.

**Table 4.** Stone-free and complications rate.

	**%**
**Stone free rate**	
1^st^ session	60.1
2^nd^ session	84.1
3^rd^ session	94.4
**Complication rate (Clavien-Dindo)**	19.7
Grade I+II	16.1
Grade III	3.6

Intraoperative and postoperative complications were grouped using the Clavien-Dindo modified classification for urological procedures. As it has been previously illustrated in Table 4, 16.1% of cases presented mild complications defined as grade I and II, and 3.6% were classified as grade III. Grades I and II were most frequently associated with an infectious-inflammatory process and mild hematuria without a significant decrease in hemoglobin levels, which remitted in less than three days after discharge. A frequent postoperative complication was also complaining about lower urinary symptoms associated with ureteral stent placement. The symptomatology disappeared in less than three days in most cases. Repositioning the double J stent under local anesthesia was classified as grade 3 according to the latest classification. No blood transfusion associated with major injuries was required during repeated surgical procedures. One patient with staghorn calculi needed prolonged double J stent placement after mucosal injuring.

During the follow-up between repeated procedures registered in the medical records of patients, there were no crossed infections associated with the endourological treatment.

## Discussion

Ectopic kidney represents a rare condition with an embryologic cause and is characterized by abnormal development of the Wolffian duct and ureteral bud of the fetus [10]. It can be found in several uncommon positions like the pelvis, thorax, abdomen, or ilium. Ectopic pelvic kidney has a reported incidence of 1 to 2200–3000 cases [2].

Anatomical abnormalities such as the position, shape, and orientation associated with the embryological maldevelopment increase the risk of further inconvenience like hydronephrosis and lithiasis, mainly caused by improper urinary drainage.

The active treatment options for renal calculi include ESWL, PNL, RIRS, laparoscopic and robotic, or even open surgery when all other options failed. However, only 1 to 2% of renal stones are treated using classical open surgical procedures [11]. The European Guidelines for urolithiasis recommend both ESWL and RIRS as the first choice in treating stones smaller than 1 cm [12]. While PNL is considered the main surgical procedure for larger calculi (>2cm), those situations with maximum dimensions between 1–2 cm are somehow placed in a grey zone.

For congenital anomalies like horseshoe kidney, ectopic pelvic kidney, and isolated rotation anomaly with associated lithiasis, the proper treatment strategy is still controversial. Since no consensus was established for these rare cases, several studies presented good results using ESWL, laparoscopy, PNL, and RIRS as the main therapeutic option. Even though it was introduced more than 30 years ago, continuous technologic development associated with increasing performance permitted flexible ureteroscopy to become an important surgical alternative for small and mid-sized calculi, even for uncommon genetic conditions. Good results using retrograde intrarenal surgery were published in recent studies [13–15].

According to the great experience of the center and low complication rates associated with flexible ureteroscopy, this technique was successfully used to treat medium and large stones with different intrarenal locations in the present study.

A good performance status associated with a low complication rate made flexible ureteroscopy a more and more preferred method being highly demanded worldwide. Several improvements referring to caliber, deflection, irrigation, and optic characteristics were made over the years [16, 17]. According to the high demand for flexible ureteroscopes, the manufacturers started developing a single-use device that already presented important advantages. Disposal ureteroscopes already proved their technical superiority regarding caliber, maximum deflection angle, and irrigation flow, while comparable results were obtained about visibility and maneuverability [18]. Another major aspect of using disposable devices is reducing crossed infections and the risk of newly installed defections during surgery. A largely debated subject is cost comparison, but some recent studies sustain the idea of comparable cost-effective characteristics since reusable scopes have to be serviced too often. Now more than five single-use scopes are available on the market. In this study, a Pusen PU3022 flexible ureteroscope was successfully used for all patients.

The stone-free rate is one of the major characteristics in evaluating the success of a procedure. Since the present study mostly enrolled patients with medium to large renal stones, in some cases, more than one intervention was needed in achieving this goal. The reported stone-free rates of 60.1% after the first session and 94.4% after the third session in complicated cases clearly present the procedure’s benefits.

In one study conducted by Bozkurt *et al.*, which analyzed the results of 26 patients diagnosed with the same pathology and treated with flexible ureteroscopy, the success rate was estimated at 84.4%. The survey also mentioned two situations with an unfavorable infundibulopelvic angle that failed for this kind of procedure [19]. Another study conducted by Ergin *et al.* aimed to compare the results of retrograde intrarenal surgery and laparoscopy on ectopic kidneys revealed comparable results for both kinds of interventions [20]. Other studies report a success rate between 75.0% to 84.7% when using flexible ureteroscopy in patients with pelvic kidneys [21, 22]. A larger review which included 86 patients with abnormal renal anatomy from 11,885 analyzed data obtained from the Clinical Research Office of the Endourological Society (CROES), revealed a decreased stone-free rate in cases with a stone burden above 80 mm^2^ [23].

Abnormal anatomy in congenital malformations can represent a major inconvenience that can lead to a failed procedure. Although methods like relocating stones using nitinol baskets from difficult positions are used, one of the most important aspects of ectopic pelvic kidneys is the ureter anatomy. Frequently, a short tortuous ureter can be a challenging situation when trying to access the intrarenal space.

In this study, the ureteral sheath was successfully placed for all cases. Only 4 cases (36.36%) required a double J stent placement for 1 to 2 weeks before intervention to avoid difficulties when placing the ureteral sheath. When faced with an abnormal ureter, a semirigid ureteroscopy was performed before placing the access sheath. Usage of the semirigid approach before the intervention when dealing with a renal malformation is also described by other authors in their studies [24, 25]. Other authors like Bozkurk described interventions performed without previous ureteral sheath placement [19]. Urgulu *et al.* suggest that a pediatric 9,5–11,5 Fr sheath may be useful to overcome the problems caused by a tortuous ureter [26].

It is generally accepted that genetic malformations of the renal anatomy may lead to a higher percentage of complication rates when performing a surgical intervention for urolithiasis compared to those performed in normal conditions. Difficulties in localizing and accessing renal stones are always expected when treating abnormal kidneys [27].

Bas *et al.* tried to identify some predictive factors related to complication rates in a retrospective study analyzing 1395 patients who underwent retrograde intrarenal surgery for renal and proximal ureteric stones. The overall complication percentage was 17.2%, while grades higher than III based on the Clavien-Dindo modified classification were encountered in only 2.4% cases [28].

In the present study, the overall complication rate was 19.7%. Clavien II and III grades were noted in 16.1% and 3.6% cases, respectively. There were no complications higher than grade III noted, which is similar to other literature findings.

The most frequently reported complications related to flexible ureteroscopy are mucosal injury (1.5%), disposition of ureteral stent (0.77%), colic (2.2%), fever and associated sepsis (1.13%), avulsion (0.1%) or strictures related to ureteral manipulation (0.1%) [29].

Since several techniques are available, a comparison of the available results obtained using methods such as ESWL, PCNL, or laparoscopy is mandatory. ESWL is the less invasive method for renal stones. Even though it presents advantages like avoiding general anesthesia, the results are often diminished by difficulties in stone localization caused by overlying bone structures or bowel gases. Abnormal anatomy also impairs urinary drainage, and fragmented stones may not be eliminated, causing a reduced stone-free rate for this procedure [27]. Ray *et al.* reported a 25% success rate after the first procedure, increasing to 63.6% after additional treatment for horseshoe kidneys [30]. After analyzing 150 cases with kidney malformations, Tunc *et al.* reported an overall stone-free rate of 68% after repeated sessions within 3 months [31]. PCNL is still considered the gold standard in treating stones bigger than 2 cm by the current guidelines. The procedure associates a high stone-free rate but also a high complication rate. The abnormal anatomy in these cases increases the risk of injuring adjoining organs [27]. After analyzing PCNL procedures on horseshoe kidneys over 15 years, Symons *et al.* revealed a 77% success rate after the first procedure [32]. Tepeler *et al.* revealed a 66.7% to 90.7% stone-free rate after repeated sessions for a mean stone of 28.4 mm [4].

One of the major limitations of the study is the small number of patients enrolled who underwent single-use flexible ureteroscopy. Since this is a rare pathology and disposable devices are relatively new, it is hard to increase the number in such a short period. Also, this is one of the first studies in this field, so there is a lack of data in the literature for comparison. Another limitation is stone composition. There were no data available in the medical records about this topic. It is well known that composition may influence the stone-free rate since hard stones like calcium oxalate correlate with a decreased percentage. Few findings on this subject are available in the literature, so further studies in this field would be helpful.

## Conclusion

Single-use flexible ureteroscopy represents a good alternative associated with a high stone-free rate and low complications when treating stones in cases with genetic malformations like an ectopic pelvic kidney. When dealing with large calculi greater than 2 cm, more than one session may be necessary to achieve a higher stone-free rate. Disposable ureteroscopes severely decrease the risk of newly appeared defections during the intervention and almost annihilate the risk of crossed infections compared to reusable ones and represent a significant step into developing a personalized treatment for each patient.

## Acknowledgments

### Ethical approval

The approval for this study was obtained from the Ethics Committee of the Sf. Ioan Emergency Clinical Hospital, Bucharest (approval ID: 9/12.04.2017).

### Consent to participate

Written informed consent was obtained from the participants.

### Conflict of interest

The authors declare that there is no conflict of interest.
